# Role of p62 nuclear condensates in regulating **ubiquitin-mediated proteasomal degradation**

**DOI:** 10.1042/EBC20253033

**Published:** 2025-11-17

**Authors:** Chen Lulu-Shimron, Aaron Ciechanover, Victoria Cohen-Kaplan

**Affiliations:** Rappaport-Technion Integrated Cancer Center (R-TICC), The Rappaport Faculty of Medicine and Research Institute, Technion-Israel Institute of Technology, Haifa, Israel

**Keywords:** LLPS condensates, p62, proteasome, protein degradation, ubiquitin

## Abstract

The ubiquitin-proteasome system (UPS) is essential for maintaining cellular proteostasis by selective proteasomal degradation of ubiquitinated proteins. Proper function of the UPS ensures turnover of proteins that have completed their role and removal of damaged proteins. Recent studies have identified p62/Sequestosome-1 as a key modulator of UPS efficiency, particularly through its ability to form dynamic, membraneless condensates via liquid-liquid phase separation. Within the nucleus, these structures recruit and concentrate components of the UPS, including its proteolytic arm — the 26S proteasome and ubiquitinated substrates. This organization enhances substrate recognition and degradation efficiency.

Nuclear p62 condensates play an essential role in controlling the turnover of oncogenic proteins. Specifically, they facilitate the proteasomal degradation of the transcription factor c-Myc and prevent its nuclear accumulation by recruiting both c-Myc and its E3 ligase complex SCF^Fbxw7^. Additionally, nuclear p62 condensates contribute to the maintenance of promyelocytic leukemia (PML) nuclear bodies and protect them from stress-induced disassembly by stabilizing the PML protein through sequestration and subsequent degradation of RING Finger Protein 4 (RNF4) — its major E3 ligase.

Under stress conditions such as oxidative stress, heat shock, or DNA damage, p62 nuclear condensates rapidly assemble and recruit molecular chaperones and ubiquitin ligases, thereby promoting the clearance of misfolded and damaged proteins. Loss of nuclear p62 or disruption of its condensate-forming domains affects UPS function and compromises proteostasis.

These findings highlight the role of p62 condensates in coordinating nuclear protein quality control and protecting cells from proteotoxic and oncogenic stress.

## Introduction

The ubiquitin-proteasome system (UPS) is vital for cellular homeostasis by mediating protein degradation and maintaining proteome integrity [1,2]. Through the selective tagging of proteins with ubiquitin (Ub) and their subsequent degradation by the 26S proteasome (a large proteolytic complex responsible for ubiquitinated proteins’ breakdown), the UPS regulates diverse cellular processes, including cell cycle progression, signal transduction, and stress responses [3,4]. This targeted degradation involves the formation of covalently attached Ub chains to internal lysine (Lys) residues of substrate proteins, marking them for recognition by the 26S proteasome [5]. Dysregulation of the UPS has been implicated in numerous pathological conditions, such as neurodegenerative diseases, cancer, and autoimmune disorders, highlighting its essential role in maintaining cellular function and health [6–9].

Ubiquitination is carried out through a highly coordinated enzymatic cascade involving the activity of three classes of enzymes: E1 ubiquitin-activating enzyme, E2 ubiquitin-conjugating enzymes (UBCs), and substrate-specific E3 Ub ligases. These enzymes act sequentially to covalently attach Ub moieties to the target proteins [10]. Protein targeting for degradation via the UPS is highly specific and tightly regulated due to the recognition of specific degradation signals in the substrates by their cognate E3s. This ensures that only proteins marked for elimination are processed [2]. However, under certain conditions, Ub-tagged proteins may evade degradation due to the activity of deubiquitinating enzymes (DUBs), which remove Ub chains from substrate proteins, thereby reversing the signal for proteasomal degradation [11]. Because of the complex structure of the Ub chains where the Ub residues are linked to one another via different internal Lys residues in the previous moiety, degradation is mediated largely by chains where the internal linkage is via Lys48. Other chains serve non-proteolytic functions, making ubiquitination a broadly used post-translational modification that serves numerous cellular functions.

The complexity of these processes raises important questions about how the participants in these multi-step reactions are recruited to the same place at the right time and how the decision is made to either degrade or retain Ub-marked proteins. Emerging evidence shows that the condensation of biomolecules via liquid–liquid phase separation (LLPS) plays an important role in the regulation of numerous biochemical reactions, including those of the UPS [12]. The formation and functionality of numerous cellular supramolecular assemblies, such as stress granules, nuclear speckles, promyelocytic leukemia (PML), and P bodies, have been shown to form via LLPS [13–16]. This review focuses on the role of nuclear p62 LLPS bodies, also known as p62 condensates, in modulating the UPS.

## p62 liquid-liquid phase separation bodies

LLPS is a physical process in which a homogeneous mixture spontaneously separates into two distinct liquid phases. This phenomenon is driven by thermodynamic conditions and weak, multivalent interactions between molecules, and it underlies the formation of various cellular structures, such as Cajal bodies, nuclear speckles, and nucleolus. These structures lack physical membranes and are defined by surface tension and chemical potential equilibrium.

The formation of LLPS-driven condensates relies on several key factors. These include high concentrations of specific macromolecules, multiple weak and transient interactions, and the presence of scaffolding proteins or proteins with intrinsically disordered regions. Additionally, other biomolecules and factors, such as RNA, post-translational modifications, and environmental changes (like temperature, pH, and salt concentration), also play an important role [17,18].

Unlike 'canonical' membrane-bound organelles, condensates are highly dynamic, with their components rapidly exchanging between the condensed and dilute phases. LLPS condensates exhibit liquid-like behavior, including the ability to flow, fuse, deform, and undergo fission under shear forces. These properties enable the reversible and highly responsive formation of bio-reactive structures, which can influence the rate of biological reactions, depending on the specific conditions [19–22]. It has been demonstrated that LLPS-driven condensation can slow biochemical reactions through various mechanisms, including the sequestration of enzymes and substrates into distinct phases, selective compartmentalization of components, and the depletion of substrates from the surrounding solution. Conversely, LLPS can accelerate reaction rates by concentrating reactants, pre-organizing enzyme–substrate complexes, and enabling rapid responses to stimuli [23].

Recent studies show that the scaffold protein p62, also known as sequestosome-1, serves as a structural basis for the formation of LLPS-mediated p62 assemblies both in cells and in cell-free systems [24]. p62 is widely distributed within cells and is found in both the cytoplasm and the nucleus. Its shuttling between the two compartments is controlled by two nuclear localization signals (NLS1 and NLS2) and a nuclear export signal (NES) [25].

**Figure 1:**
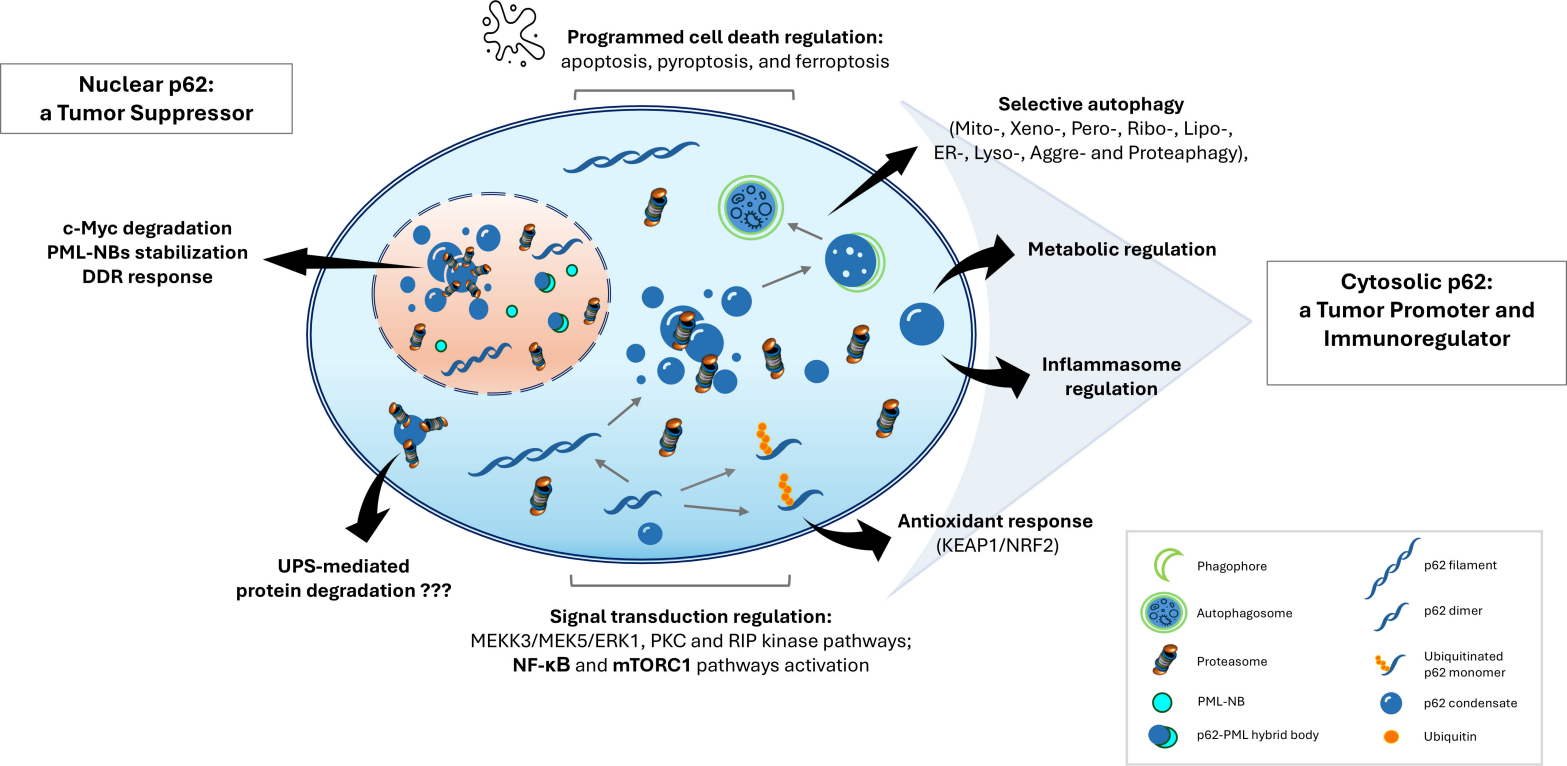
Cytosolic and nuclear functions of p62. p62 is a multifunctional adaptor protein that participates in numerous cellular pathways impacting health and disease. **In the cytosol**, p62 regulates diverse signaling including NF-κB, mTOR, and KEAP1/NRF2 pathways, activates mitogen-activated protein kinase (MAPK) and other stress-responsive cascades, mediates selective autophagy by targeting protein aggregates, pathogens, and damaged organelles for lysosomal degradation, influences cellular metabolism, and modulates different forms of programmed cell death. Cytosolic p62 is also suggested to contribute to ubiquitin–proteasome system (UPS)-mediated degradation. **In the nucleus**, p62 facilitates proteasomal degradation of specific substrates, such as c-Myc and RNF4, supports the stabilization of PML-NBs, and is involved in maintaining genome integrity through regulation of DNA damage responses (DDR). PML-NB, promyelocytic leukemia nuclear bodies.

p62 is involved in various cellular processes, including activation of signaling and metabolic pathways, such as NF-κB inflammatory signaling, mTORC1, NRF2, apoptosis, adipogenesis, and autophagy (Figure 1) [26–32]. It contains several functional domains that contribute to its diverse roles, among them: (i) the ubiquitin-associated (UBA) domain, which binds polyubiquitin chains; (ii) LC3-interacting region, which facilitates interactions with autophagy machinery; (iii) Kelch-like ECH-associated protein 1 (KEAP1)-interacting region (KIR), which regulates oxidative stress responses; and (iv) N-terminal Phox and Bem1 (PB1) domain, which mediates interactions with numerous key signal transduction molecules and the proteasome [33,34].

A novel aspect of p62’s functionality is its ability to undergo phase separation, resulting in the formation of dynamic structures known as p62 condensates. These condensates are formed through two parallel but independent intermolecular interactions: (i) p62 oligomerization via its PB1 domain which leads to the formation of helical filaments, and (ii) its interaction with ubiquitinated proteins via its C-terminal UBA domain. p62 oligomerization enhances its affinity for cargo, directing proteins for degradation [35].

The formation of p62 filaments is driven by the interaction between a Lys residue in position 7 of the first p62 molecule and an aspartate (Asp) residue in position 69, which resides in the PB1 domain of the second one [36]. This Lys7-Asp69 interaction provides the structural basis for p62 polymerization, allowing the protein to form extended filaments [37]. Substitution of Lys7 with Arginine, as well as PB1 depletion, prevents p62 polymerization and its subsequent condensation [36]. However, despite these changes, p62 can still bind to ubiquitinated proteins through its UBA domain [34].

Recently, it has been shown that p62 condensates act as a platform for autophagosome biogenesis by coordinating the recruitment of proteins essential for autophagosome nucleation (FIP200, ULK1, ATG13, and ATG101) [38], membrane gathering (ATG9 and ATG16L1-positive vesicles, which serve as membrane sources for phagophore assembly), and enzymatic activities of the ULK1 complex, which mediates PI3P generation [39]. This coordination leads to the subsequent recruitment of autophagy-related proteins, such as LC3, which are essential for the expansion of the autophagosomal membrane and the progression of autophagy [40].

Under certain pathophysiological conditions, such as disease-associated mutations, environmental stress, aging, or post-translational modifications, initially dynamic, liquid-like condensates can gradually mature into gel-like or solid aggregates. This transition is driven by the strengthening intermolecular interactions and conformational changes in proteins, leading to the formation of stable β-sheet–rich fibrils that are characteristic of amyloid aggregates [41]. Specifically, excessive stabilization or crosslinking of p62 oligomers impairs molecular exchange, driving their transition toward a gel-like or solidified state. Also, the scaffolding protein SHKBP1 reduces p62 condensate mobility and droplet dynamics. Once formed, these aggregates become stiff, irreversible, and persistent, thereby disrupting normal cellular dynamics and may contribute to different pathologies [42].

Maintaining the liquidity of p62 condensates is essential for their physiological function. Balanced interactions with polyubiquitin chains and post-translational modifications, such as phosphorylation at Ser403 of p62, promote dynamic, reversible binding without triggering aggregation. Another mechanism involves autophagy-mediated elimination of ‘aged’ condensates [43]. Also, the cytoskeleton modulates local p62 concentration and facilitates both efficient assembly and disassembly of droplets [44].

## Nuclear p62 condensates: how they differ from other phase-separated nuclear assemblies

p62-containing nuclear condensates function as quality control hubs that mediate the proteasomal degradation of abnormal proteins and proteins that have completed their function following their modification by Ub. These structures are fundamentally distinct from other membraneless nuclear organelles formed via LLPS, such as Cajal bodies and nuclear speckles, both in composition and function. Cajal bodies, characterized by the marker protein coilin, serve as sites for the assembly and maturation of small nuclear ribonucleoproteins and other RNA-processing complexes [45], whereas nuclear speckles act as storage and modification centers for pre-mRNA splicing factors, including SC-35, and contribute to splicing regulation at active transcription sites [46].

Occasional partial colocalization of p62 with Cajal bodies has been observed, particularly under conditions of proteotoxic stress. Such interactions are likely transient and context-dependent, reflecting a role for p62 in the clearance of ubiquitinated proteins in proximity to RNA-processing compartments, rather than a direct involvement in RNA metabolism. Notably, p62 is not detected in nuclear speckles, underscoring its functional segregation from these RNA-based organelles [25].

## Role of p62 condensates in modulation of UPS activity

One of the fundamental questions in regulating protein degradation via the UPS is how the reactants in this multistep process are spatially organized to accelerate reaction rates and respond rapidly to changing pathophysiological conditions. Growing evidence suggests that reactants are recruited to specific cellular compartments by mediators that act as scaffolds, facilitating efficient reactions [20]. One such mediator is p62, which is a scaffold protein. Due to its multiple interaction domains, p62 has been shown to recruit all necessary components for the ubiquitination reaction, including substrate proteins, thereby regulating protein degradation via the UPS.

**Figure 2:**
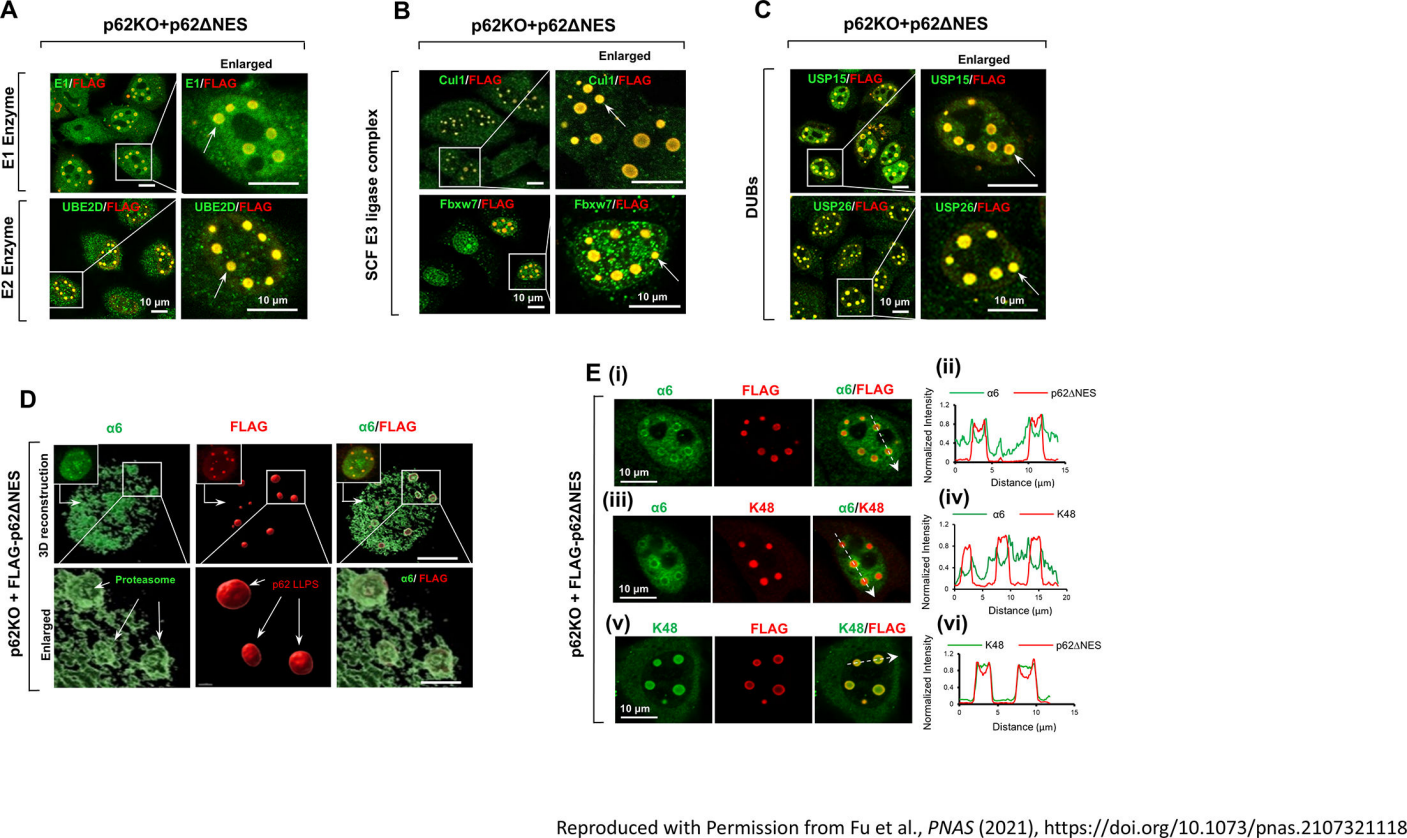
Nuclear p62 condensates recruit components of the ubiquitin-proteasome system (UPS). **(A**) E1 and E2s (UbcH5A/B/C). (**B**) Components of the SCF E3 ligase complex - Cullin 1 (CUL1) and the F-box protein Fbxw7. (**C**) DUBs - USP15 and USP26. White arrows point to the condensates that contain the different components. (**D**) The proteasome. Three-dimensional reconstruction of confocal z-stacks showing localization of the proteasome (the α6 subunit) at the periphery of nuclear p62 condensates, forming an organized outer layer. White arrows point to the p62 condensates. (**E**) Ubiquitin chains and the proteasome. Colocalization profiles of α6, K48-linked ubiquitin chains, and FLAG-p62ΔNES: (**i**, **iii**, and **v**). Intensity tracings along the dashed arrows in panels **i**, **iii**, and **v**, showing α6, p62, and K48-linked ubiquitin chains, respectively (**ii**, **iv**, and **vi**). SCF, S-phase kinase-associated protein 1-CUL1-F-box.

**Figure 3:**
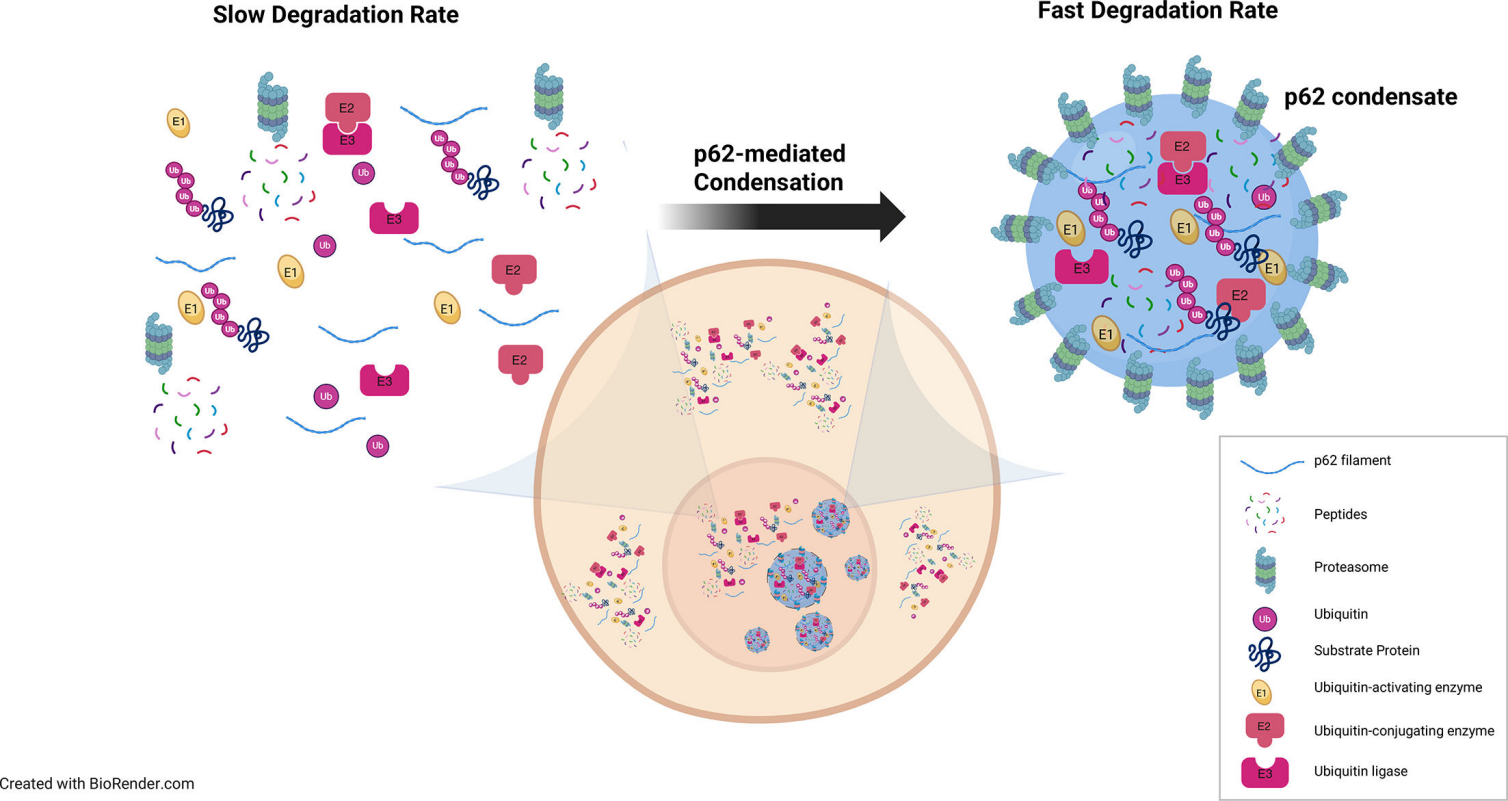
Schematic model illustrating enhanced UPS-mediated proteolysis within nuclear p62 condensates. Degradation of nuclear proteins can be accelerated when all components of the UPS are concentrated within nuclear p62 condensates. Notably, proteasomes are organized into an outer layer surrounding the p62 condensates, potentially optimizing substrate accessibility and degradation efficiency.

The engagement of p62 condensates in proteasome-mediated degradation has been demonstrated using a p62 species expressed solely in the nucleus (p62∆NES), which is not involved in the proteolytic activity of the autophagy-lysosome system. A recent study shows that p62 condensates recruit E1, E2s (Figure 2A), E3s (Figure 2B), and DUBs (Figure 2C) along with a proteolytically active 26S proteasome (Figure 2D), and target substrates (Figure 3) [34–36]. This spatial co-clustering at high concentrations of all components creates a favorable microenvironment for efficient protein degradation.

Interestingly, the 26S proteasome has been shown to form an outer layer surrounding p62 condensates (Figure 2D and 2Ei-ii), whereas the ubiquitinated proteins and the UPS components reside within the core of the condensate (Figure 2A–C and 2Eiii-vi). This arrangement suggests that the ubiquitination reaction and protein catalysis occur in distinct regions within the same condensate.

Importantly, it has been shown that nuclear p62 condensates accumulate substrate proteins marked by at least two types of Ub chains — K48- and K63-linked [24,30,31]. K63-linked Ub chains have previously been associated with p62-mediated targeting of cargo proteins and entire organelles for degradation via autophagy [10,47,48]. However, the role of K48-linked chains within p62 condensates, primarily known as recognition signals for proteasomal degradation [49], has remained elusive.

To address this enigma, the possible role of proteasomal degradation of K48-linked ubiquitinated proteins within p62 condensates was studied using NLS-GFP-CL1 as a model protein [30]. In this construct, the NLS directs the GFP-fusion protein to the nucleus, while the CL1-degron destabilizes it, making GFP a substrate for proteasomal degradation. The study demonstrated that under basal conditions, nuclear p62 condensates significantly enhance the proteasomal degradation of this fusion protein, effectively functioning as active centers for proteasomal proteolysis. This finding adds yet another layer to the 'canonical' role of p62 in cytoplasmic autophagy, highlighting its novel function in facilitating proteasome-mediated protein degradation.

## Role of nuclear p62 condensates in degradation of c-Myc

p62 is a multifunctional protein involved in regulating various vital cellular processes. In most studied cases, p62 was reported to be localized in the cytosol, where its roles in autophagy regulation, mTOR activation, oxidative stress response, and tumor progression are well established [28,29,50]. However, several studies have demonstrated that p62 localization in tumors is not restricted to the cytoplasm, and it can be found also in the nucleus. Notably, the presence of nuclear p62 in tumor cells has been correlated with improved patient survival [51,52], suggesting a distinct and potentially protective role for nuclear p62 in cancer biology.

**Figure 4:**
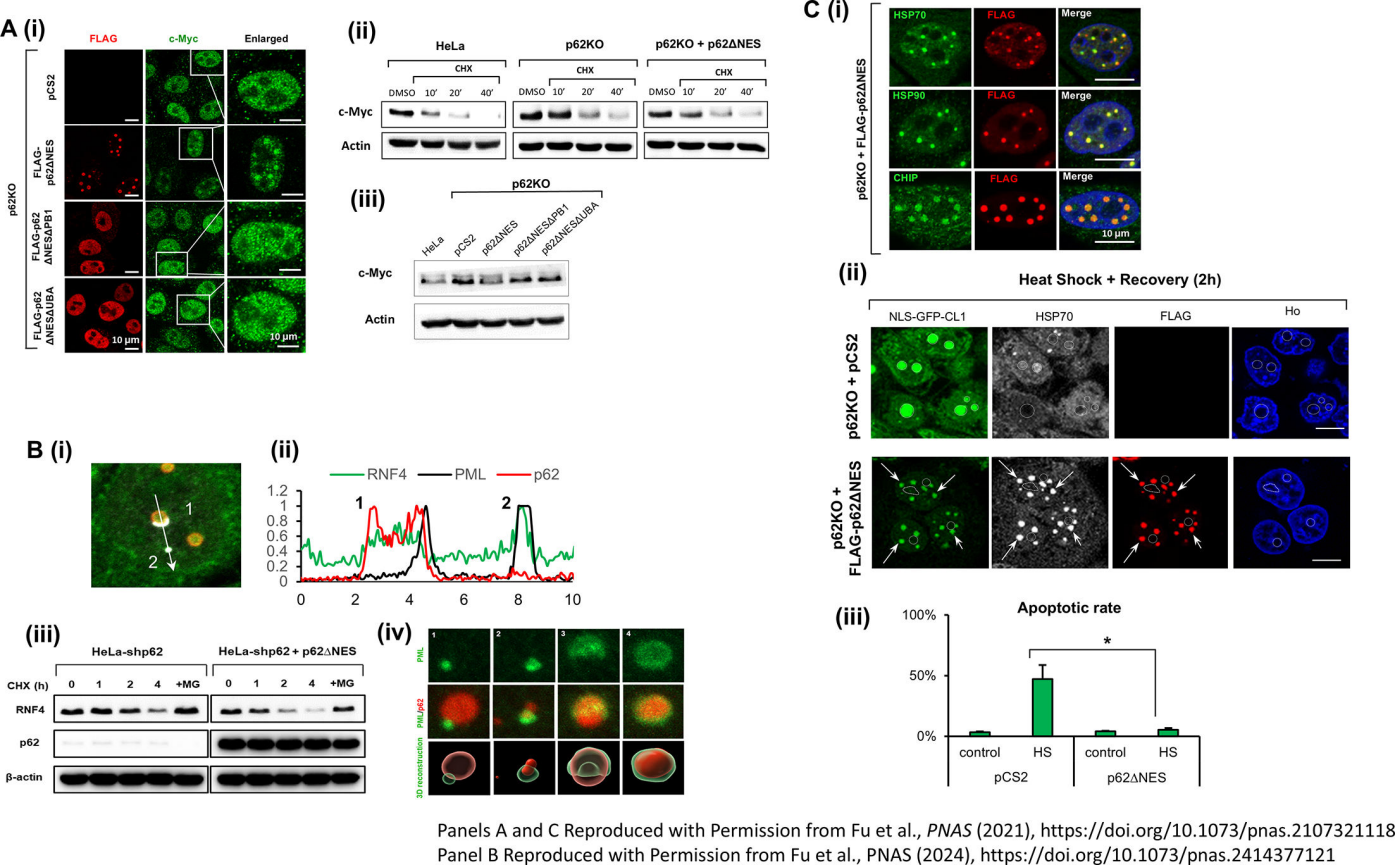
Nuclear p62 condensates modulate degradation of UPS targets and coordinate nuclear protein quality control (PQC). **(A**) Nuclear p62 condensates promote the degradation of c-Myc. (**i**) Immunofluorescence imaging showing the distribution of endogenous c-Myc within the nucleus upon overexpression of the different p62 species in p62KO HeLa cells; (**ii**) Cycloheximide (CHX) chase showing the degradation of c-Myc at the indicated time points, in the presence or absence of nuclear p62. c-Myc degradation is slower in the p62KO cells compared with wildtype HeLa and p62ΔNES-expressing cells; (**iii**) Steady-state levels of endogenous c-Myc in p62KO cells expressing the indicated mutants of p62. (**B**) Nuclear p62 condensates stabilize PML-NBs by capturing RNF4 and promoting its proteasomal degradation. (**i**) A representative fluorescence image showing RNF4 localization within both p62 condensate with anchored PML-NB (1) and with free PML-NB (2); (**ii**) Intensity profiles measured along the dashed arrows in panel (**i**). The data suggest that the interaction between PML-NBs and p62 condensates results in the redistribution of RNF4 (1), and it is now largely concentrated within the attached p62 condensate; (**iii**) Monitoring proteasomal degradation of RNF4 in the presence or absence of nuclear p62 in HeLa cells using a CHX chase; (**iv**) 3D reconstruction of super-resolution imaging of FLAG-p62ΔNES and endogenous PML in p62KO HeLa cells. Immunofluorescence staining reveals four distinct stages of the interaction between p62 condensates and PML-NBs: (1) docking, (2) capturing, (3) partial encapsulation, and (4) complete encapsulation of p62 within PML-NBs. (**C**) Nuclear p62 condensates recruit components of the PQC machinery and mitigate stress-induced toxicity. (**i**) Immunofluorescence staining shows heat shock protein 70 (HSP70), heat shock protein 90 (HSP90), and the E3 ligase C-terminus of Hsc70-Interacting Protein (CHIP) localization within p62ΔNES condensates; (**ii**) Nuclear p62 condensates prevent heat shock-induced accumulation of NLS-GFP-CL1 fusion protein in nucleoli by mediating its recruitment. Dashed circles indicate nucleoli, and arrows point to p62 condensates; (**iii**) Measurement of heat stress-induced apoptosis in p62KO and p62KO + p62ΔNES HeLa cells after 24 h recovery. Apoptotic cells were detected by immunofluorescence staining for cleaved caspase-3.

Recent research has shown that under steady-state conditions, nuclear p62 forms condensates that facilitate proteasome-mediated degradation of c-Myc, a well-known oncogenic transcription factor (Figure 4A) [30]. c-Myc regulates the expression of genes involved in cell cycle progression, metabolism, and cell survival, and its aberrant stabilization is strongly associated with the development and progression of various cancers [53]. When p62 function is compromised (whether due to mutations, impaired ability to form condensates, or complete absence in cells - p62KO) the degradation of c-Myc is significantly reduced, leading to its pathological accumulation (Figure 4A) [30]. The recruitment of c-Myc into p62 condensates is thought to increase its local concentration, thereby enhancing its accessibility to E3 Ub ligases responsible for its ubiquitination and subsequent degradation. Immunofluorescence staining has shown that endogenous c-Myc colocalizes with p62 nuclear condensates, which also overlap with the SCF [S-phase kinase-associated protein 1 (SKP1)–Cullin 1 (CUL1)–F-box] E3 Ub ligase complex - SCF^FBXW7^ [30]. The SCF complex is essential for targeting a variety of nuclear oncoproteins for degradation. It comprises three core components: (i) CUL1, which acts as a scaffold; (ii) SKP1, which links CUL1 to F-box proteins; and (iii) RING-box protein 1, which recruits the E2 enzymes. That, alongside a variable substrate recognition component, is the F-box protein [54]. Among these, FBXW7 specifically recognizes c-Myc and targets it for degradation as part of the SCF complex. The presence of FBXW7 within these condensates further supports the role of nuclear p62 in regulating the degradation of c-Myc and other nuclear oncoproteins. This regulation is critical, as excessive c-Myc expression can drive unchecked cell division and tumorigenesis [55].

These findings reveal a novel function of nuclear p62, underscoring its important role in controlling the expression of c-Myc and probably related nuclear oncoproteins, thereby contributing to tumor suppression and the maintenance of cellular homeostasis.

## Role of p62 in degradation of unincorporated proteasomal subunit

An additional important question that remains unresolved is how cells maintain the proper stoichiometry of proteasomal components and prevent the accumulation of potentially harmful excess subunits. The recent study [30] showed that unincorporated proteasomal subunits, such as β4 and Rpn11, are removed by the proteasome. Moreover, p62 condensates have been shown to play a crucial role in their degradation via the UPS. It has been shown that p62 condensates recruit and degrade overexpressed β4- and Rpn11-GFP. Importantly, the distribution of unincorporated subunits within p62 condensates differs from that of fully assembled proteasomes. While the entire 26S proteasome 'decorates' the p62 condensates, unincorporated β4- and Rpn11-GFP subunits localize to the core of the condensates, similar to other substrate proteins. Interestingly, only a portion of the overexpressed unincorporated subunits can be found within the p62 condensate core. Their recruitment is dependent on their ubiquitination which is recognized by p62’s UBA domain. It is possible that the entire cohort (or almost all) of the unassembled subunits are degraded within the condensates, and what we see is a frozen time point along a dynamic process. In the context of proteasomal regulation, p62’s ability to facilitate the degradation of individual proteasomal subunits underscores its role in maintaining the balance of the UPS machinery itself, further extending its importance in cellular quality control (see also below on the more general role of the condensates in maintaining the protein quality control PQC).

## p62 stabilizes promyelocytic leukemia nuclear bodies

Another important function of p62-containing nuclear condensates is their role in stabilizing promyelocytic leukemia nuclear bodies (PML-NBs) [31]. PML-NBs are dynamic, membraneless nuclear structures that play key roles in various essential cellular processes by recruiting a range of nuclear proteins to coordinate genome maintenance, DNA repair, cellular responses to stress and viral infection [56,57]. Additionally, PML-NBs contribute to both tumor suppression and apoptosis by serving as a platform for the activation and post-translational modification of key proteins such as p53, which regulate apoptosis [58]. Loss or dysfunction of PML-NBs has been associated with impaired apoptotic pathways and increased tumorigenesis [59].

Under both physiological conditions and stress such as occurs after exposure to arsenic trioxide that promotes PML-NBs degradation [60], nuclear p62 condensates function to maintain the structural integrity of PML-NBs [31]. This protective effect is mediated through the selective sequestration of the E3 Ub ligase RNF4 (Figure 4B i–ii), which targets the PML protein for proteasomal degradation (Figure 4B iii). By capturing RNF4 within p62 condensates and facilitating its accelerated degradation, p62 effectively protects PML-NBs from disassembly.

The molecular mechanism that underlies this process involves a specific interaction between the proline-rich domain of the PML protein and the PB1 domain of p62. This interaction facilitates the docking of PML-NBs onto p62 condensates and promotes the formation of a shell-like arrangement of PML-NBs surrounding the p62 condensates (Figure 4B, iv). Notably, these hybrid structures (p62-PML bodies) remain spatially distinct and do not undergo fusion or content mixing, suggesting a tightly regulated compartmentalization [31]. This spatial organization allows for the selective sequestration of RNF4 while maintaining the functional independence of the two distinct structures [31].

The stabilization of PML-NBs by p62 is of fundamental biological importance because PML-NBs play important roles in tumor suppression. By inhibiting RNF4-mediated ubiquitination and the subsequent proteasomal degradation of PML, p62 condensates help preserve both the structural integrity and functional activity of PML-NBs. This protective mechanism may enhance the tumor-suppressive functions of PML-NBs, which include activating p53 signaling, inducing apoptosis, and promoting cellular senescence. As a result, the nuclear functions of p62 extend beyond its traditional roles in autophagy, representing a novel regulatory axis in the cell’s defense against oncogenic stress and tumorigenesis.

## The role of p62 condensates in regulation of stress response and PQC

Various cellular stress conditions, including heat shock, oxidative stress, and DNA damage, can induce structural alteration in proteins, potentially leading to proteotoxicity and triggering cell death. Under these conditions, the UPS becomes essential for the integrity of the cellular proteome by facilitating the rapid degradation of aberrant proteins [10].

The studies discussed above suggest that nuclear p62 condensates act as dynamic hubs for proteasome-mediated degradation during cellular stress. In contrast to autophagy, which operates on a relatively slower time scale, the UPS enables rapid and efficient clearance of misfolded/damaged proteins. This process is significantly enhanced by the stress-induced formation of nuclear p62 condensates, which amplify the cellular stress response by increasing the number of proteolytically active sites [30].

These condensates selectively sequester ubiquitinated misfolded or damaged proteins while concurrently recruiting key components of the PQC machinery, including molecular chaperones such as heat shock protein 70 (HSP70) and heat shock protein 90 (HSP90), and E3 Ub ligases such as C-terminus of Hsp70-interacting protein (CHIP) (Figure 4C i) [31]. Through this coordinated assembly, p62 proteolytic condensates facilitate the efficient recognition and proteasomal degradation of aberrant proteins, thereby reducing the accumulation of stress-induced aggregates and maintaining nuclear proteostasis (Figure 4C ii).

Importantly, p62 nuclear condensates also prevent the accumulation of misfolded proteins within the nucleolus (Figure 4C ii), a vital organelle responsible for ribosome biogenesis and which is highly sensitive to proteotoxic stress. By promoting the degradation of nuclear damaged proteins and preserving nucleolar integrity, p62 condensates help to mitigate the harmful effects of damaged protein accumulation in these compartments and reduce cell death (Figure 4C iii) [30].

## Summary

Nuclear p62 condensates have emerged as dynamic proteolytic hubs within the nucleus, playing crucial roles in PQC through the UPS. They facilitate the degradation of nuclear proteins, including oncogenic factors such as c-Myc, by accelerating their ubiquitination and subsequent proteasomal degradation and contribute to proteostasis by removing unassembled proteasomal subunits to maintain stoichiometric balance in multi-subunit complexes. In addition, nuclear p62 condensates interact with and stabilize PML-NBs, an established tumor suppressor, by sequestering and promoting degradation of RNF4, its Ub E3 ligase, which otherwise would have targeted PML for proteolysis. This stabilization suggests that nuclear p62 condensates may act indirectly as tumor suppressors by enhancing PML-NB integrity and function.

The discovery of nuclear p62 condensates adds a significant new layer to the cellular quality control network, integrating protein degradation, oncogene regulation, and tumor suppressors’ protection under a single hub. Important future investigations should determine whether analogous condensates exist in the cytosol and define their roles in proteostasis and tumorigenesis/tumor suppression. Key questions remain about the mechanisms governing substrate selection and recruitment, the dynamic interplay with other nuclear bodies, and the regulatory signals controlling condensate assembly and activity under physiological and stress conditions. Addressing these questions through advanced *in vivo* imaging, spatial proteomics, and functional genomics will deepen our understanding of p62 condensate biology. Ultimately, such insights may usher in the development of novel therapeutic modalities modulating the activity of p62 condensates.

**Summary points**

- Nuclear p62 condensates — and possibly similar cytosolic counterparts — have been identified as hubs facilitating protein degradation through the ubiquitin–proteasome system (UPS).
- The role of the nuclear condensates in removal of an oncogene (c-Myc) and stabilization of a tumor suppressor (promyelocytic leukemia nuclear bodies) raises the possibility that they themselves should be defined as tumor suppressors.
- The condensates were also shown to remove excess of unassembled proteasomal subunits, which probably also applies to a broader role in balancing the stoichiometry in multi-subunit complexes.
- Altogether, the discovery of nuclear p62 condensates’ function has added yet another layer to the complex networks of cellular quality control machineries.

## Open Access

This article has been published open access under our Subscribe to Open programme, made possible through the support of our subscribing institutions, learn more here: https://portlandpress.com/pages/open_access_options_and_prices#conditional

## Abbreviations

ATG: autophagy-related protein
Asp: aspartate
CUL1: Cullin 1
DDR: DNA damage response
DUB: deubiquitinating enzymes
ERK1: Extracellular signal-regulated kinase 1
FIP200: FAK family-interacting protein of 200 kDa
LLPS: liquid–liquid phase separation
Lys: lysine
MAPK: mitogen-activated protein kinase
MEK5: Mitogen-activated protein kinase kinase 5
MEKK3: Mitogen-activated protein kinase kinase kinase 3
NES: nuclear export signal
NLS: nuclear localization signal
NRF2: nuclear factor erythroid 2-related factor 2
PB1: Phox and Bem1
PI3P: phosphatidylinositol 3-phosphate
PKC: protein kinase C
PML: promyelocytic leukemia
PML-NBs: promyelocytic leukemia nuclear bodies
PQC: protein quality control
RIP: Receptor-interacting protein
SCF: S-phase kinase-associated protein 1–Cullin 1–F-box
SKP1: S-phase kinase-associated protein 1
UBA: ubiquitin-associated
UBC: ubiquitin-conjugating enzyme
ULK1: Unc-51-like kinase 1
UPS: ubiquitin–proteasome system
Ub: ubiquitin
